# Editorial: Reviews in breast cancer

**DOI:** 10.3389/fonc.2023.1161583

**Published:** 2023-05-11

**Authors:** Maria Rosaria De Miglio, Claudia Mello-Thoms

**Affiliations:** ^1^ Department of Medicine, Surgery and Pharmacy, University of Sassari, Sassari, Italy; ^2^ Department of Radiology, The University of Iowa, Iowa City, IA, United States

**Keywords:** breast cancer, diagnosis, prognosis, education, anxiety, artificial intelligence

According to the GLOBOCAN there were an estimated 19.3 million new cancer cases and 10 million cancer deaths worldwide in 2020 (1). Female breast cancer (BC) has surpassed lung cancer and today it is the most diagnosed type of cancer (2.3 million new cancer cases, representing 11.7% of all cancer cases). In terms of mortality, it ranks in 5^th^ place, with 685,000 deaths in 2020. For women, BC represents 1 in 4 cancer cases and accounts for 1 in 6 cancer deaths (1). Moreover, the GLOBOCAN Cancer Tomorrow prediction tool estimates that incidence will increase by more than 46% by 2040 (2). However, incidence rates are not equal around the world. They are 88% higher in developed countries than in developing countries (55.9 vs. 29.7 per 100.000 women, respectively), but mortality rates are 17% higher in developing countries compared to developed countries (15.0 vs. 12.8 per 100.000 women, respectively). There are a number of reasons for the higher incidence rates in the developed countries, including early age at menarche, later age at menopause, advanced age at first birth, fewer number of children, in addition to lifestyle factors such as obesity, physical inactivity and alcohol intake.

Incidence of BC rapidly increased in the 1980s and 1990s, but by the 2000s incidence had dropped or stabilized. However, since 2007 there has been a slow increase of BC incidence of 0.5% per year in the United States, and moderate increases have been reported in several countries in Europe and in Oceania (2). Using cancer registry data, supplemented with tumor marker information to further understand these increases in incidence, it has been found that most breast cancers are estrogen-receptor positive (1). This particular type of cancer is associated with the obesity epidemic and with mammography screening, which tends to detect slow growing cancers like estrogen-receptor positive cancers. The analysis has also shown that incidence rates are falling for estrogen-receptor negative cancers (1).

Five-years survival rates range between 85-90% for developed countries, whereas for developing countries, particularly those located in Africa, it is 66%. This is primarily due to late-stage presentation of the disease, which reflects on the lack of screening programs and weak health infrastructure. As a result, mortality rates in Africa are among the world’s highest (1).

In this Special Issue our focus was to bring some state-of-the-art research in breast cancer to light. In here the reader will find papers on prognostic and potential therapeutic factors, such as immune cells in the tumor microenvironment, inflammations, small extracellular vesicles, RNA binding proteins, dysbiosis, etc. Moreover, social factors will also be discussed, such as the risk of anxiety, depression and sexual disfunction, as well as health-related quality of life in BC patients. The effects of tobacco smoking and breast cancer risk will also be explored. In terms of breast cancer diagnosis, we will examine the diagnostic value of multiple ultrasound techniques, as well as the role of Artificial Intelligence. We will also explore the use of educational tools to improve radiologists’ performance when detecting this disease. Finally, we will discuss the recent progress of therapeutic vaccines for breast cancer.

A growing body of evidence demonstrated a relationship between inflammation and cancer. It increases the risk of cancer development influencing occurrence and progression (3). IL-6 triggers chronic inflammation and cancer, it was higher in many solid tumors including BC (4) which correlated with poor prognosis and metastasis (5). As summarized by Chen et al. several antibodies for IL-6/IL-6R have been used, either as single drug or combined with chemotherapy, demonstrating a marked outcome in both preclinical and clinical trials. IL-6/JAK/STAT3 pathway suppresses anti-tumor immune responses in BC tumor microenvironment. Therefore, treatments against this pathway have given benefit for patients with BC by reducing tumor cell growth and stimulating anti-tumor immunity. Combining IL-6 pathway inhibitor with other targets therapies may represent a new strategy to treat human cancers.

The most important cause of BC death is disease progression due to metastases. Because of this challenge, the identification of unambiguous molecular biomarkers to predict the disease response is needed. Wang et al. conducted a meta-analysis assessing that higher CD68+ and CD163+ tumor-associated macrophages (TAM) density, accounting for approximately 50% of tumor microenvironment cells, is associated with poor outcome in BC patients and also with higher tumors size, no vascular invasion, and positive ER expression, highlighting the significant prognostic value for TAMs in BC patients.

The triple negative breast cancer (TNBC) is the most aggressive and invasive BC subtype, with rapid progression, short response duration to available treatment and poor clinical outcomes. Therefore, there is an urgent need to develop new early diagnosis tools and therapies with good efficacy. Zhou et al. summarized the role of small extracellular vesicles (sEVs) in TNBC. sEVs are natural nano-sized extracellular vesicles with lipid membrane outside and bioactive contents inside, produced by nearly all cell types, play a significant role in intercellular communications. sEVs contribute to angiogenesis, immune escape, tumor proliferation, invasion and distant metastasis, and drug resistance in TNBC. sEVs can be simply detected in body fluids. So, they hold great promise as biomarkers for early diagnosis, prognosis and treatment approach of TNBC. Huertas-Caro et al. argued that higher levels of tumor infiltrating lymphocytes (TILs) in TNBC have been associated with better outcomes and a higher rate of pathological complete response to neoadjuvant chemotherapy. Similar results were observed for CD4+, CD8+ TILs, independently to the human population analyzed. All together these results suggest that TILs subpopulations might have a prognostic role in TNBC, although the underlying mechanism demands to be elucidated.

Cancer stem cells are a small population of cancer cells with self-renewal and differentiation potential, responsible for tumor heterogeneity, recurrence, metastasis and drug resistance (6). Xu et al. reviewed that breast cancer stem cells (BCSCs) obtained from the same tumor exhibit heterogeneity in terms of mutations, transcriptional programs, immune characteristics and functional properties. Therefore, BCSC concept not only has extensive and great implications for cancer biology, but also has strongly clinical significance for the development of personalized therapies.

RNA binding proteins (RBPs) are key regulators of RNA metabolism. mRNAs as unstable and degradable biomacromolecules bind to specific RBPs and form complexes to maintain their stability in cells, within which RBPs control their localization, stability, translation, and degradation binding to specific mRNAs regions (7). Presently, functional inactivation or abnormal expression of RBPs may be closely associated with BC development, which means that RBPs may become good diagnostic and prognostic biomarkers for BC. Chen et al. described the role of several RBPs and their target genes in the BC development and progression, as well as Lu et al. summarized the function of RBPs in BC cells and their regulatory mechanisms. The RBPs role in drug resistance is still little know and can become a new research direction. Although, as described by Chen et al. therapeutic strategies are developing against RBPs, as the inhibition of HuR by KH-3 that blocks the invasion of BC cells by destroying the HuR-FOXQ1 mRNA interaction, the compound ZM-32 that prevent the formation of HuR-RRM1/2–VEGFA mRNA complex suppressing proliferation, migration, growth, and angiogenesis of BC cells.

Zhang et al. discussed about the emergent role of gastrointestinal microbiome as an important player in the risk and progression of BC. Supposing that the treatment of gut microbiota to stabilize the microenvironment may decrease the production and propagation of pro-tumorigenic factors and determining new approaches to stabilize these deleterious fluctuations is of interest in the treatment and prognosis of BC.

Zhang et al. provided a meta-analysis to evaluate the prognostic differences between multicentric/multifocal (MM) and unifocal BC, in order to illustrate a theoretical basis for the design of an applicable therapeutic strategy for treating MMBC patients. However, MMBC patients showed a higher death risk, but it may not be independently associated with poorer outcomes. MMBC and UFBC patients with appropriate surgery and adjuvant therapies showed the same prognosis, although the prognostic impact of every lesion in MMBC still needs further investigation.

Lei et al. summarized that germline BRCA1/2 mutations are common in Chinese patients with hereditary breast, ovarian, prostate and pancreatic cancers. Although Chinese consensuses recommend BRCA1/2 genetic testing for cancer patients only, depending on cost-effectiveness and social and political factors, public interest and patients’ benefits. The Authors recommended that healthy individuals harboring pathogenic mutations should be identified to promote prevention, early diagnosis, and timely treatment of BRCA mutation-related cancers, which may increase 5-year survival for these patients.

Social factors that affect breast cancer patients were also discussed. For example, anxiety and depression risk in Taiwanese women with breast cancer and women with cervical cancer was explored by Yang et al. As they compared these two populations of patients, the authors found that they are both at an elevated likelihood of developing anxiety and depression, but that the risk for developing depression was slightly higher in breast cancer patients.

In addition, sexual dysfunctions in breast cancer patients were examined by Hernandez-Blanquisett et al.. The authors report that up to 75% of women treated for breast cancer report sexual disorders, but oncologists are not trained to recognize which patients are at high risk for developing this disease. The authors suggest that the choice of less toxic treatments in the surgical, chemotherapy and radiation therapy domains could lead to a reduced risk of female sexual dysfunction without increasing the risk for breast cancer recurrence or the effectiveness of treatment.

In another meta-analysis and systematic review, Chen et al. studied the health-related quality of life in breast cancer patients in Asia. The authors reported that Asian breast cancer patients suffer from poor quality of life and were severely impacted by the effects of fatigue and hair loss, pain, insomnia, and anxiety.

Also in this Research Topic, He et al. carried out a systematic review and meta-analysis on the relationship between tobacco and breast cancer. They showed that active or passive smoking increased the risk of BC in women, and that the effect of smoking was influenced by factors such as duration, intensity, number of years since quitting, as well as population-related factors (such as fertility status) and breast cancer subtypes.

In terms of breast cancer diagnosis, Li et al. explored the diagnostic value of multiple ultrasound techniques for assessment of lymph node metastases in breast cancer patients. As the authors posit, early diagnosis of lymph node metastases is very important for prognosis of breast cancer development. Currently the most commonly used method is lymph node biopsy, however it is an invasive method that may bring complications to the patients (such as lymphedema). The authors found that the combination of ultrasound with contrast-enhanced ultrasound led to the best performance among all the ultrasound techniques tested.

Moreover, the use of Artificial Intelligence for the diagnosis and prognosis prediction of breast cancer was explored by Jones et al. In their review the authors focused on two tasks (1): better understanding the association between radiomics features and tumor microenvironment; and (2) the progress developing new computer-assisted aid schemes for predicting breast cancer risk, determining the likelihood of tumor malignancy, and determining tumor response to treatment.

Aiming to improve radiologists’ performance when detecting early BC, Trieu et al. explored the use of an educational intervention, BREAST (Breastscreen REader Assessment STrategy), which helps radiologists’ interpretation skills when reading both mammograms and Digital Breast Tomosynthesis cases. The authors described the use of the BREAST platform in countries with screening programs for breast cancer (such as Australia, Singapore) and countries without (such as China, Vietnam).

The recent progress on the development of therapeutic vaccines for BC has been explored by Zhang et al. in this issue. Although advanced BC is still considered to be a poorly immunogenic disease, the great success of cancer immunotherapy is paving the way for a new era in cancer treatment. Vaccine targets have included both tumor-associated antigens and tumor-specific antigens. However, as only a few women seem to benefit from neoantigens, more attention is being paid to overexpressed antigen-based treatments, such as HER-2-derived peptide vaccines.

Finally, Lyu et al. have determined the research trends and hot spots of breast cancer management during the COVID-19 pandemic. The authors suggest that during the epidemic the management of breast cancer patients changed considerably, including all aspects of management such as screening, treatment, follow-up and rehabilitation.

## Conclusions

Breast cancer is currently the most diagnosed type of cancer for women worldwide. Moreover, the GLOBOCAN Cancer Tomorrow estimates that incidence of this disease will increase by more than 46% by 2040, making it critical that we device new ways to detect, diagnose and treat breast cancer.

In this Special Issue we presented reviews and meta-analyses that promoted knowledge of the mechanisms of breast cancer progression, as well as its prevention, diagnosis and treatment. We believe that this information will be useful for both scientists and clinicians.

## Author contributions

Paper design: CMT. Writing: MRdM and CMT. Revisions: CMT. All authors read and agreed to the submitted version of the manuscript.
